# Astragalus polysaccharide inhibits the development of urothelial carcinoma by activating AMPK signaling to induce BENC1-xCT complex formation

**DOI:** 10.18632/aging.205007

**Published:** 2023-09-20

**Authors:** Guangquan Tong, Xiaowei Wang, Shuangfeng Chen, Yanyang Jin

**Affiliations:** 1 Department of Urology, The First Affiliated Hospital of Jinzhou Medical University, Jinzhou, Liaoning 121001, China; 2 Department of Otorhinolaryngology Head and Neck Surgery, The First Affiliated Hospital of Jinzhou Medical University, Jinzhou, Liaoning 121001, China; 3 Department of Surgery, Golmud Second People’s Hospital, Haixi Mongolian and Tibetan Autonomous Prefecture, Golmud, Qinghai 816099, China

**Keywords:** astragalus polysaccharides, ferroptosis, urothelial carcinoma, AMPK activation, BECN1-xCT complex

## Abstract

In recent years, the incidence of urothelial carcinoma (UC) has been high in men. The aim of this study was to investigate whether astragalus polysaccharide (APS) could inhibit the development of UC and the specific molecular mechanism. Our data showed that APS inhibited the proliferation of UC cells in a dose-dependent manner, and APS reduced the migratory capacity of RT4 and T24 cells. Further studies revealed that the ferroptosis inhibitor ferrostatin-1 (Fer-1) reversed APS-induced cell death, intracellular Fe2+ and malondialdehyde (MDA) accumulation, and lipid peroxidation product deposition. The Western blot and immunofluorescence results showed that APS significantly inhibited the expression of glutathione peroxidase 4 (GPX4) but did not alter the protein level of solute carrier family 7 member 11 (xCT, SLC7A11). Further analysis revealed that APS reduced the activity of xCT in RT4 and T24 cells. Moreover, APS significantly increased the phosphorylation levels of protein kinase AMP-activated catalytic subunit alpha 1 (AMPK) and BECN1 in RT4 and T24 cells, which induced the formation of the BECN1-xCT complex. However, when AMPK was silenced in RT4 and T24 cells, APS-induced ferroptosis was reversed to some extent, indicating that APS-mediated ferroptosis involves AMPK signaling. Moreover, APS has been shown to inhibit tumor growth in nude mice *in vivo*. In summary, our study demonstrated for the first time that APS could promote the formation of the BECN1-xCT complex in UC cells by activating AMPK/BECN1 signaling, which inhibited the activity of xCT to reduce GPX4 expression, thereby inducing ferroptosis and ultimately inhibiting UC progression.

## INTRODUCTION

Bladder cancer (BC) is the ninth most commonly diagnosed tumor in the world, and the incidence of BC in men far exceeds that in women [1]. Urothelial carcinoma (UC) is the most common pathological type of bladder cancer and was formerly known as metastatic cell carcinoma of the bladder, accounting for more than 90% of all cases of bladder cancer [2]. UC is characterized by rapid development and a high recurrence rate; the 5-year survival rate of patients is only 6%, and the prognosis is very poor [3]. UC mainly includes nonmuscle invasive bladder cancer (NMIBC) and muscle invasive cancer (MIBC) [1]. Approximately 25% of all patients diagnosed with UC exhibit metastatic urothelial carcinoma (mUC) or NMIBC [4, 5]. According to reports, mUC is a fatal disease, with a 5-year overall survival rate (OS) of approximately 15% [4, 5]. Therefore, radical surgery is not suitable for these patients [4]. Thus, improving and prolonging the overall survival rate of mUC patients is an important clinical and scientific issue.

Ferroptosis is a novel cell death pathway associated with intracellular free iron overload-induced lipid peroxidation [6, 7]. Under normal conditions, membrane-bound sodium-dependent cystine/glutamate reverse transporter (System Xc-), which is composed of light chain subunits (xCT, SLC7A11) and heavy chain subunits (SLC3A2), works together with glutathione peroxidase 4 (GPX4) to regulate the synthesis of reduced glutathione (GSH), which reduces lipid peroxidation and prevents ferroptosis [7, 8]. In contrast, ferroptosis occurs when the expression or activity of System Xc- and GPX4 is inhibited in response to ferroptosis inducers such as salazosulfapyridine and sorafenib [9, 10]. Various studies have shown that antitumor drugs can induce ferroptosis in tumor cells [11, 12]. For instance, in breast cancer cells, metformin reduces the protein stability of xCT by inhibiting the UFMylation process, thereby triggering ferroptosis [11]. In addition, baicalin can reduce the expression of ferritin heavy chain 1 (FTH1), thus promoting the release of Fe2+ to induce ferroptosis and inhibiting the progression of BC [12]. Recent studies have found that the System Xc- inhibitor erastin induces ferroptosis in tumor cells followed by increased protein expression levels of BECN1, a key regulator of autophagy [13, 14]. Further studies revealed that BECN1 binds directly to xCT to form a complex that inhibits xCT activity, which is largely dependent on AMPK-mediated phosphorylation of BECN1 at serines 90, 93 and 96 [13, 14]. After overexpression of BECN1 in human mesenchymal stem cells and extraction of the corresponding exosomes, this BECN1-enriched-exo could inhibit xCT-driven GPX4 expression thereby inducing ferroptosis to ameliorate liver fibrosis [15]. Inhibition of the BECN1-xCT complex attenuates lipid peroxidation and enhances antioxidant capacity after early brain injury in rats, thereby improving neurological dysfunction [16]. These studies confirm that silencing BECN1 reduces the formation of the BECN1-xCT complex thereby attenuating ferroptosis.

Astragalus has tumor suppressive effects, and its important active ingredient Astragalus polysaccharide (APS) has attracted much attention in the field of oncology because of its ability to kill tumors directly, indirectly or synergistically with chemotherapy [17, 18]. In addition to antitumor effects, APS has other effects, such as immune modulation and antioxidant and hypoglycemic effects, and demonstrates good clinical application prospects [18]. However, whether APS can inhibit the development of UC and the specific underlying mechanism have not been reported. For the first time, we investigated the effects and molecular mechanisms of APS-mediated inhibition of UC *in vivo* and *in vitro*, thus laying the foundation for the clinical application of APS to treat UC.

## RESULTS

### APS inhibits the proliferation and migration of RT4 and T24 cells

**Figure 1:**
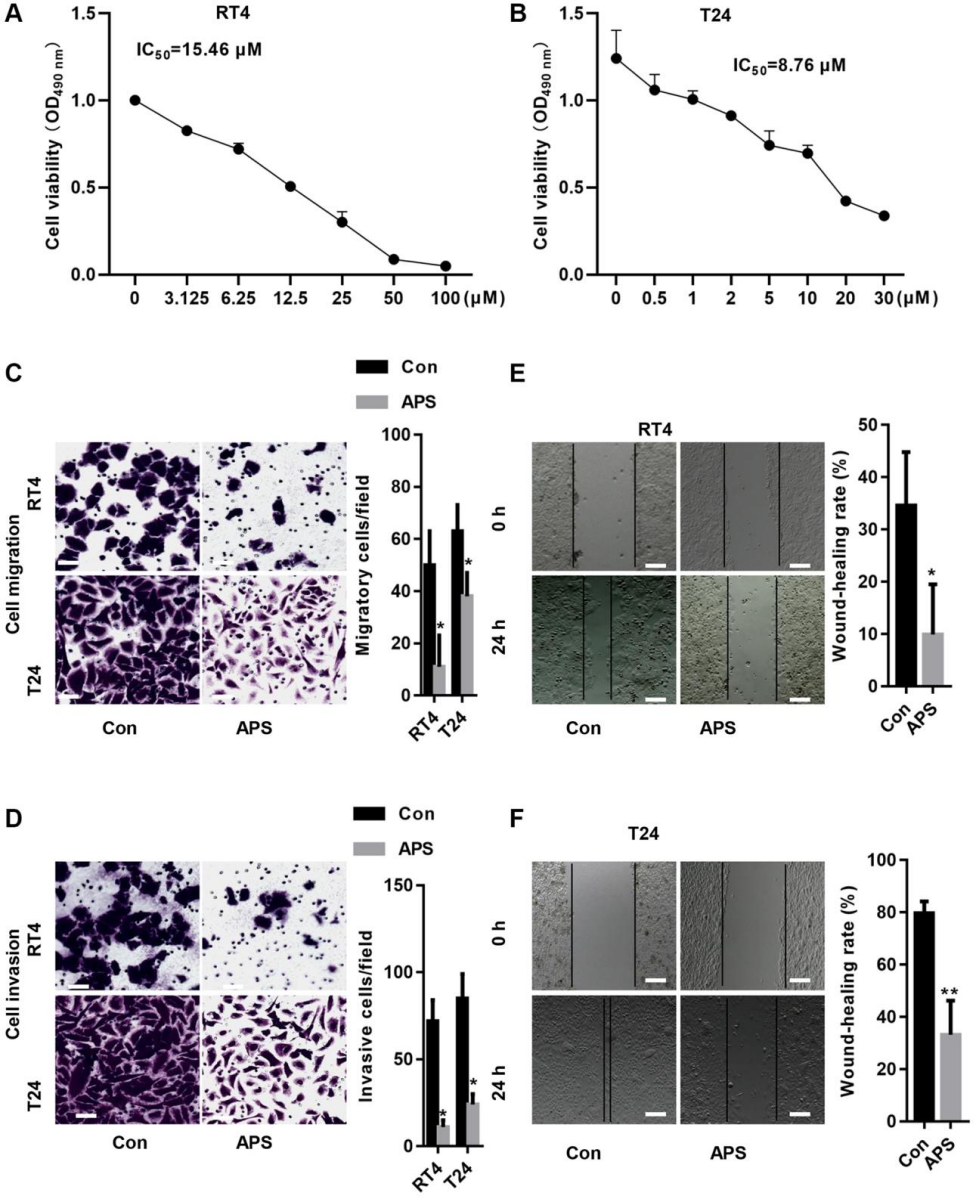
**APS inhibits the proliferation and migration of RT4 and T24 cells.** CCK-8 assays revealed that APS significantly inhibited the viability of RT4 (**A**) and T24 (**B**) cells. The transwell results revealed that 15 and 10 μM APS significantly inhibited the migration of RT4 (**C**) and T24 (**D**) cells (Bar represents 10 μm, 20 × magnification). Scratch assays confirmed that 15 and 10 μM APS significantly reduced the migration of RT4 (**E**) and T24 (**F**) cells (Bar represents 50 μm, 4 × magnification). ^*^p < 0.05, ^**^p < 0.01 vs. Con.

First, we treated RT4 and T24 cells with different concentrations of APS for 24 h. The CCK-8 results revealed that the IC50 values of APS in RT4 and T24 cells were 15.46 and 8.76 μM, respectively (Figure 1A, 1B). Next, we treated RT4 and T24 cells with 15 and 10 μM APS for 24 h. The Transwell results revealed that 15 and 10 μM APS significantly inhibited the migration of RT4 and T24 cells (Figure 1C, 1D). Similarly, scratch assays confirmed that 15 and 10 μM APS significantly reduced the migration of RT4 and T24 cells (Figure 1E, 1F).

### APS induces ferroptosis in RT4 and T24 cells

**Figure 2:**
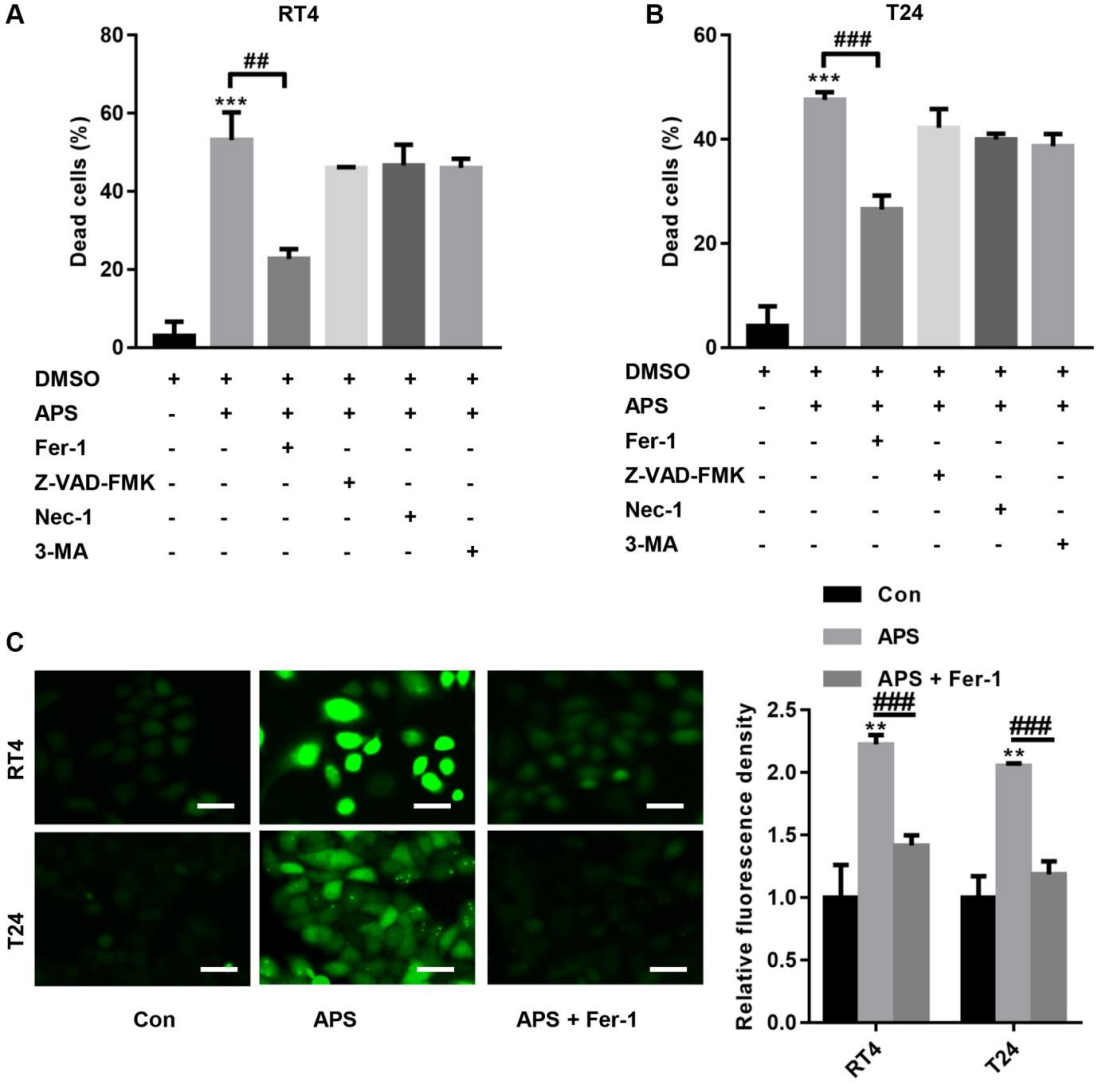
**APS induces ferroptosis in RT4 and T24 cells.** RT4 and T24 cells were treated with 1 μM ferrostatin (Fer-1, a ferroptosis inhibitor), 20 μM Z-VAD-FMK (a pancaspase inhibitor), 20 μM necrostatin-1 (Nec-1, a necrosis inhibitor) and 10 μM 3-methyladenine (3-MA, an autophagy inhibitor). Then, RT4 and T24 cells were treated with 15 and 10 μM APS for 24 h. The CCK-8 results showed that Fer-1 significantly reversed APS-induced death in RT4 (**A**) and T24 (**B**) cells. (**C**) C11 BODIPY 581/591 staining revealed that Fer-1 reversed APS-induced lipid peroxidation in RT4 and T24 cells (Bar represents 20 μm, 20 × magnification). ^**^p < 0.01, ^***^p < 0.001 vs. Con; ^##^*p* < 0.01, ^###^*p* < 0.001 vs. APS.

We further examined the mechanism by which APS inhibits the proliferation and migration of RT4 and T24 cells. First, RT4 and T24 cells were treated with 1 μM ferrostatin (Fer-1, a ferroptosis inhibitor), 20 μM Z-VAD-FMK (a pancaspase inhibitor), 20 μM necrostatin-1 (Nec-1, a necrosis inhibitor) and 10 μM 3-methyladenine (3-MA, an autophagy inhibitor). Then, RT4 and T24 cells were treated with 15 and 10 μM APS for 24 h. The CCK-8 results showed that 15 and 10 μM APS significantly increased the mortality rates of RT4 and T24 cells (Figure 2A, 2B). In contrast, Fer-1 significantly reversed APS-induced death in RT4 and T24 cells (Figure 2A, 2B). Furthermore, we found that APS significantly increased lipid peroxidation in RT4 and T24 cells, while Fer-1 reversed APS-induced lipid peroxidation in RT4 and T24 cells (Figure 2C).

### Fer-1 reverses APS-induced ferroptosis

**Figure 3:**
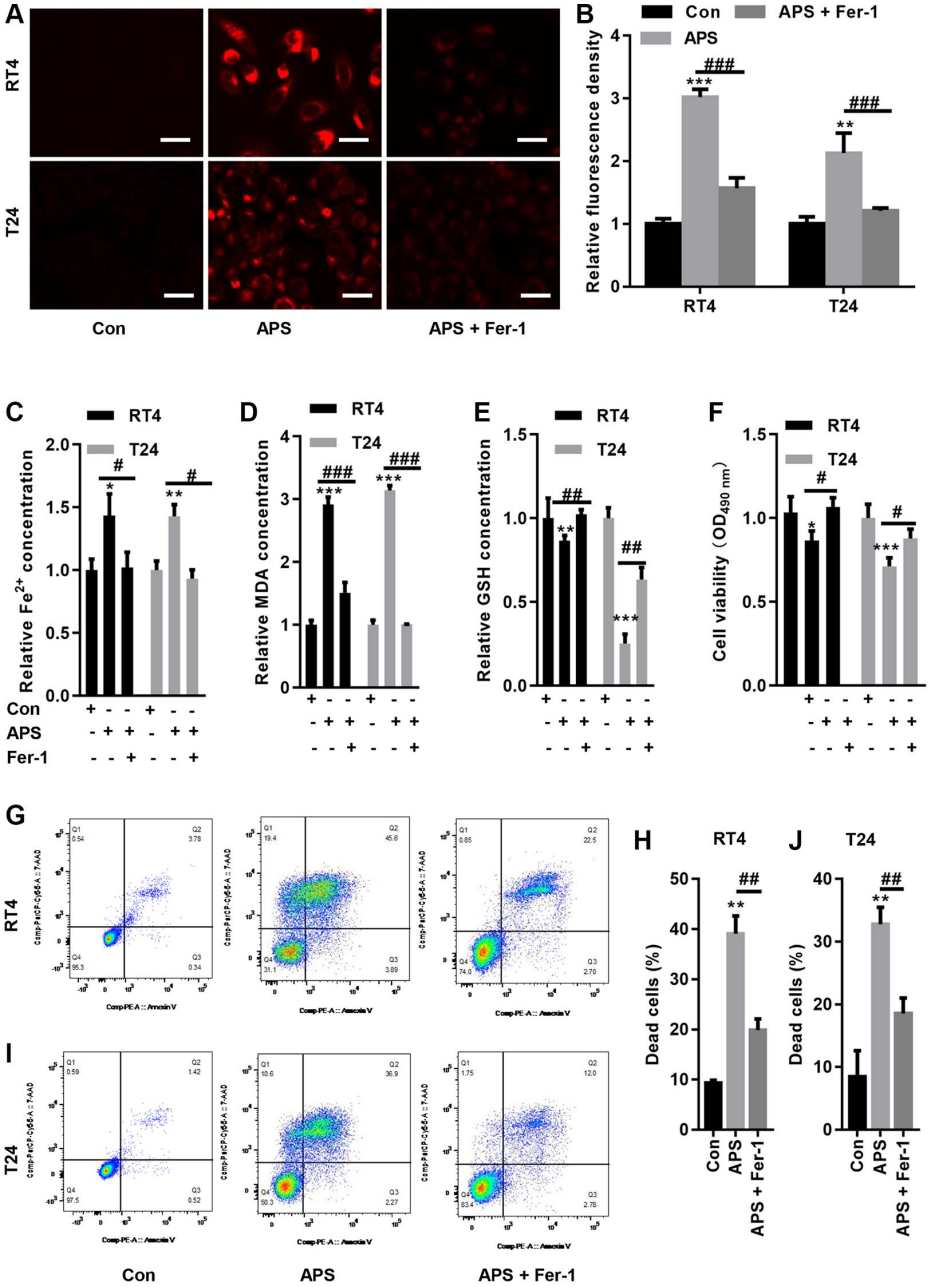
**Fer-1 reversed APS-induced ferroptosis.** (**A**) FerroOrange staining showed that Fer-1 could reduce the APS-induced increase in Fe^2+^ levels (Bar represents 20 μm, 20 × magnification). (**B**) Statistical analysis showed that Fer-1 reversed APS-induced Fe^2+^ (**C**) and MDA (**D**) accumulation in RT4 and T24 cells. Fer-1 reversed the APS-induced decrease in GSH levels (**E**) in RT4 and T24 cells. (**F**) CCK-8 analysis showed that the APS-induced decrease in the viability of RT4 and T24 cells could also be ameliorated by Fer-1. Flow cytometry revealed that Fer-1 treatment reduced the APS-induced increase in the mortality of RT4 (**G**, **H**) and (**I**, **J**) T24 cells. ^*^p < 0.05, ^**^p < 0.01, ^***^p < 0.001 vs. Con; ^#^*p* < 0.05, ^##^*p* < 0.01, ^###^*p* < 0.001 vs. APS.

We examined Fe^2+^ levels in RT4 and T24 cells. APS significantly increased Fe^2+^ accumulation in RT4 and T24 cells, while Fer-1 significantly reduced the APS-induced increase in Fe^2+^ levels (Figure 3A–3C). Moreover, APS increased the lipid oxidation product MDA, while Fer-1 reversed APS-induced MDA accumulation (Figure 3D). In contrast, APS reduced GSH levels in RT4 and T24 cells. Conversely, Fer-1 significantly antagonized the APS-induced decrease in GSH levels (Figure 3E). Additionally, the APS-induced decrease in the viability of RT4 and T24 cells could be improved by Fer-1 (Figure 3F). Flow cytometry revealed that APS increased the mortality of RT4 and T24 cells, while Fer-1 treatment reduced the increase in cell mortality caused by APS (Figure 3G–3J).

### APS inhibits xCT activity and reduces GPX4 expression

**Figure 4:**
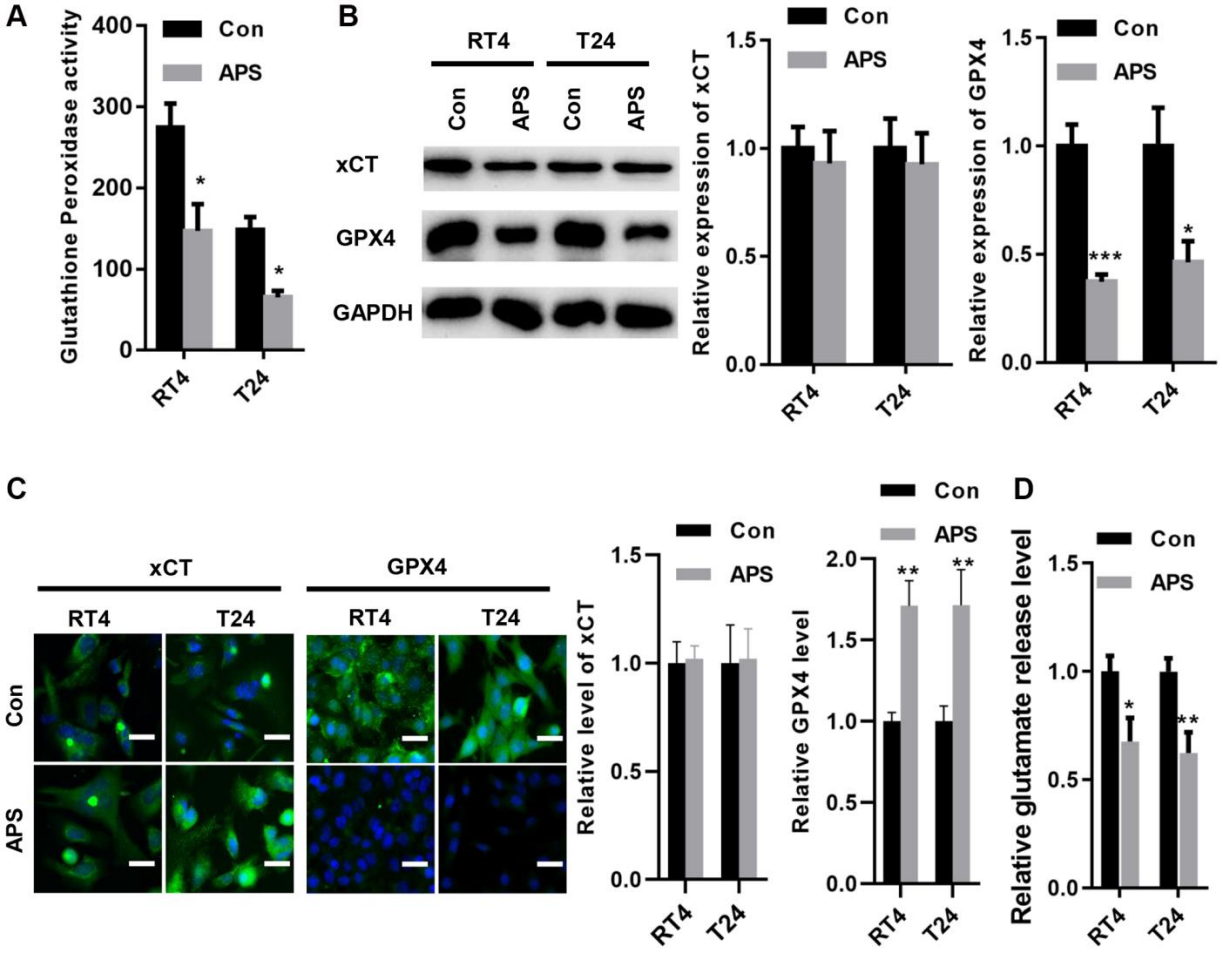
**APS inhibited the activity of xCT and reduced the expression of GPX4.** (**A**) APS significantly reduced the activity of glutathione peroxidase in RT4 and T24 cells. (**B**) The Western blot results revealed that APS did not change the expression of xCT in RT4 and T24 cells but reduced the protein level of GPX4. (**C**) The IF results showed that APS did not change the fluorescence level of xCT but significantly inhibited the fluorescence expression of GPX4 (Bar represents 10 μm, 20 × magnification). (**D**) APS reduced the activity of xCT in RT4 and T24 cells. ^*^p < 0.05, ^**^p < 0.01, ^***^p < 0.001 vs. Con.

In RT4 and T24 cells, APS significantly reduced the activity of glutathione peroxidase (Figure 4A). Moreover, the Western blot results revealed that APS did not alter xCT expression in RT4 and T24 cells but reduced GPX4 protein levels (Figure 4B). The IF results showed similar outcomes. xCT fluorescence levels in RT4 and T24 cells were not altered by APS, but GPX4 fluorescence expression was significantly inhibited (Figure 4C). Although APS did not alter the protein expression of xCT, APS reduced the activity of xCT, as evidenced by a decrease in glutamate release (Figure 4D).

### APS activates AMPK/BECN1 signaling in RT4 and T24 cells

**Figure 5:**
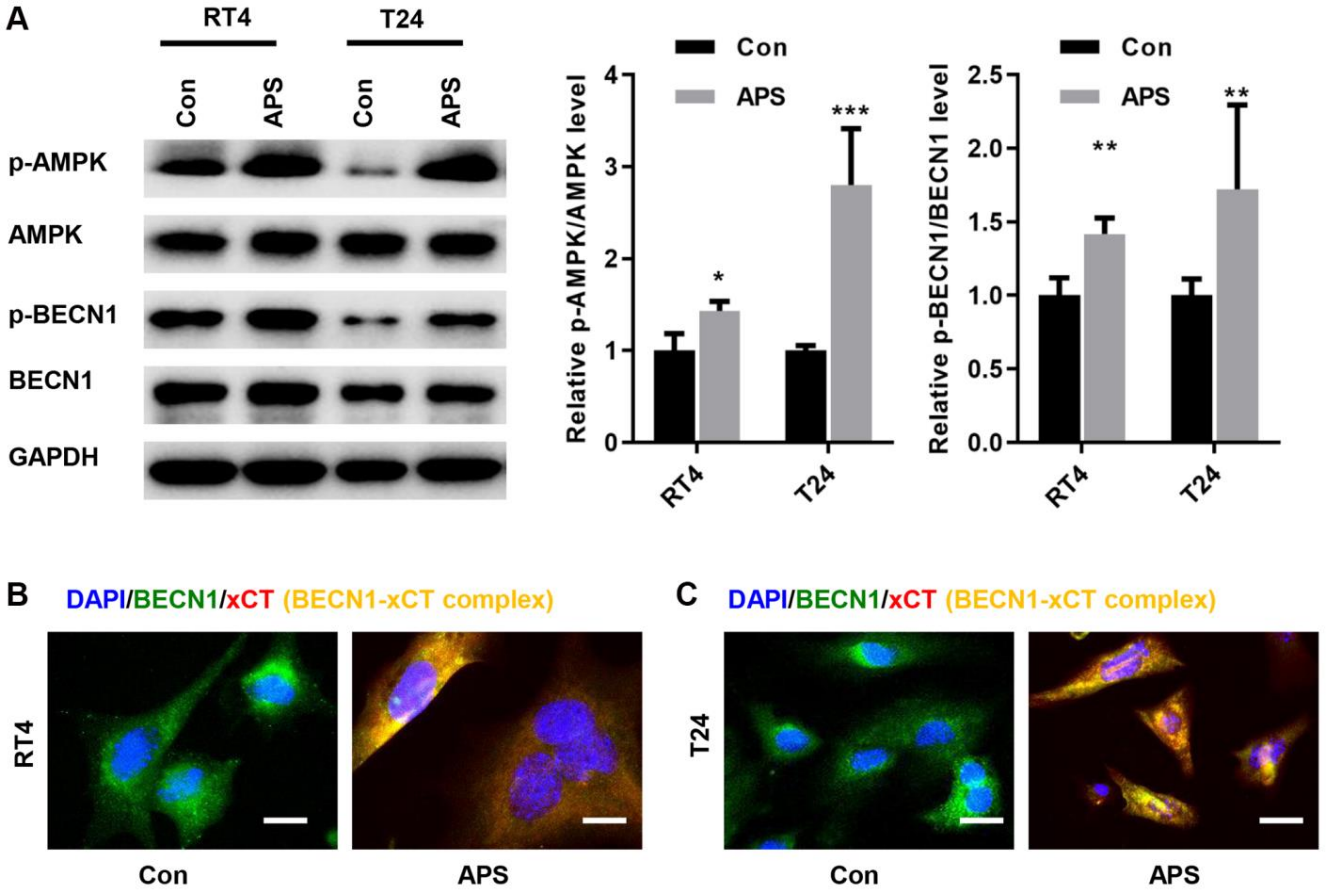
**APS activates AMPK/BECN1 signaling in RT4 and T24 cells.** (**A**) The Western blot results showed that APS significantly increased the phosphorylation levels of AMPK and BECN1 in RT4 and T24 cells. IF staining showed that APS induced the formation of the BECN1-xCT complex in RT4 (**B**) and T24 (**C**) cells (Bar represents 10 μm, 40 × magnification). ^**^p < 0.01, ^***^p < 0.001 vs. Con.

A previous study showed that AMPK could phosphorylate BECN1, thereby forming a BECN1-xCT complex that inhibits xCT activity and promotes ferroptosis [13]. Therefore, we examined whether APS activated AMPK/BECN1 signaling in RT4 and T24 cells. In RT4 and T24 cells, APS significantly increased the phosphorylation levels of AMPK and BECN1 (Figure 5A). Further studies revealed the formation of the BECN1-xCT complex in RT4 and T24 cells after APS treatment, as evidenced by enhanced yellow fluorescence (Figure 5B, 5C).

### Knockdown of AMPK reverses APS-induced ferroptosis

**Figure 6:**
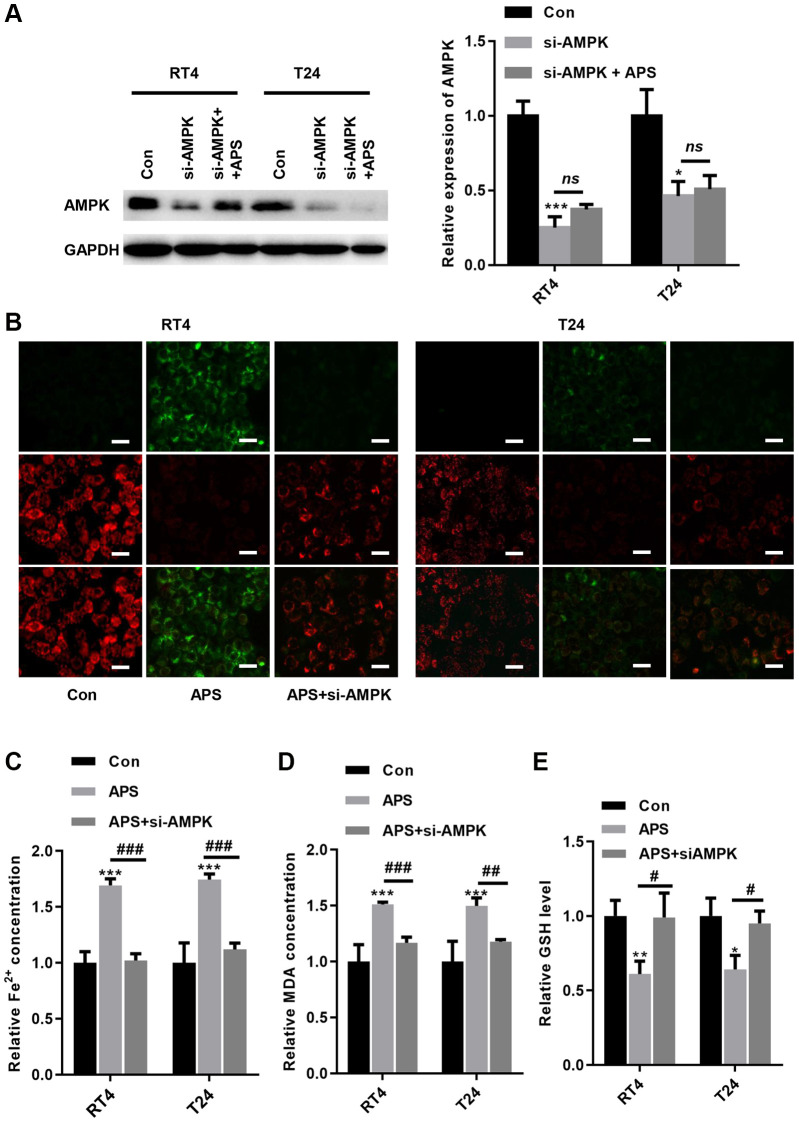
**Knockdown of AMPK reverses APS-induced ferroptosis.** (**A**) The Western blot results showed that the transfection of si-AMPK significantly inhibited AMPK expression even in APS-treated RT4 and T24 cells. (**B**) the JC-1 results showed that silencing AMPK significantly ameliorated APS-induced upregulation of MMP (Bar represents 20 μm, 20 × magnification). In RT4 and T24 cells, the APS-induced increase in Fe^2+^ (**C**) and MDA (**D**) levels could be alleviated by si-AMPK to some extent. (**E**) In RT4 and T24 cells, the reduction of GSH induced by APS could be reversed by knockdown of AMPK. ^*^p < 0.05, ^***^p < 0.001 vs. Con; ^##^*p* < 0.01, ^###^*p* < 0.001 vs. APS.

To further verify whether APS-induced ferroptosis was achieved via AMPK signaling, we transfected siRNA targeting AMPK into RT4 and T24 cells. The Western blot results revealed that si-AMPK transfection significantly inhibited AMPK expression in RT4 and T24 cells, even in APS-treated RT4 and T24 cells (Figure 6A). We observed the mitochondrial membrane potential (MMP) changes in mitochondria in RT4 and T24 cells by JC-1 staining. Compared with those in the Con group, APS increased MMP in RT4 and T24 cells, but treatment with si-AMPK reversed such enhancement (Figure 6B). In contrast, silencing AMPK significantly reduced APS-induced MMP upregulation (Figure 6B). Moreover, the APS-induced increase in Fe^2+^ and MDA levels could be alleviated by si-AMPK to some extent (Figure 6C, 6D). Meanwhile, APS decreased GSH levels in RT4 and T24 cells, but knockdown of AMPK could alleviate the reduction of GSH levels (Figure 6E). Accordingly, we hypothesize that APS-induced ferroptosis in RT4 and T24 cells is mediated by AMPK.

### APS inhibits tumor growth in nude mice *in vivo*

**Figure 7:**
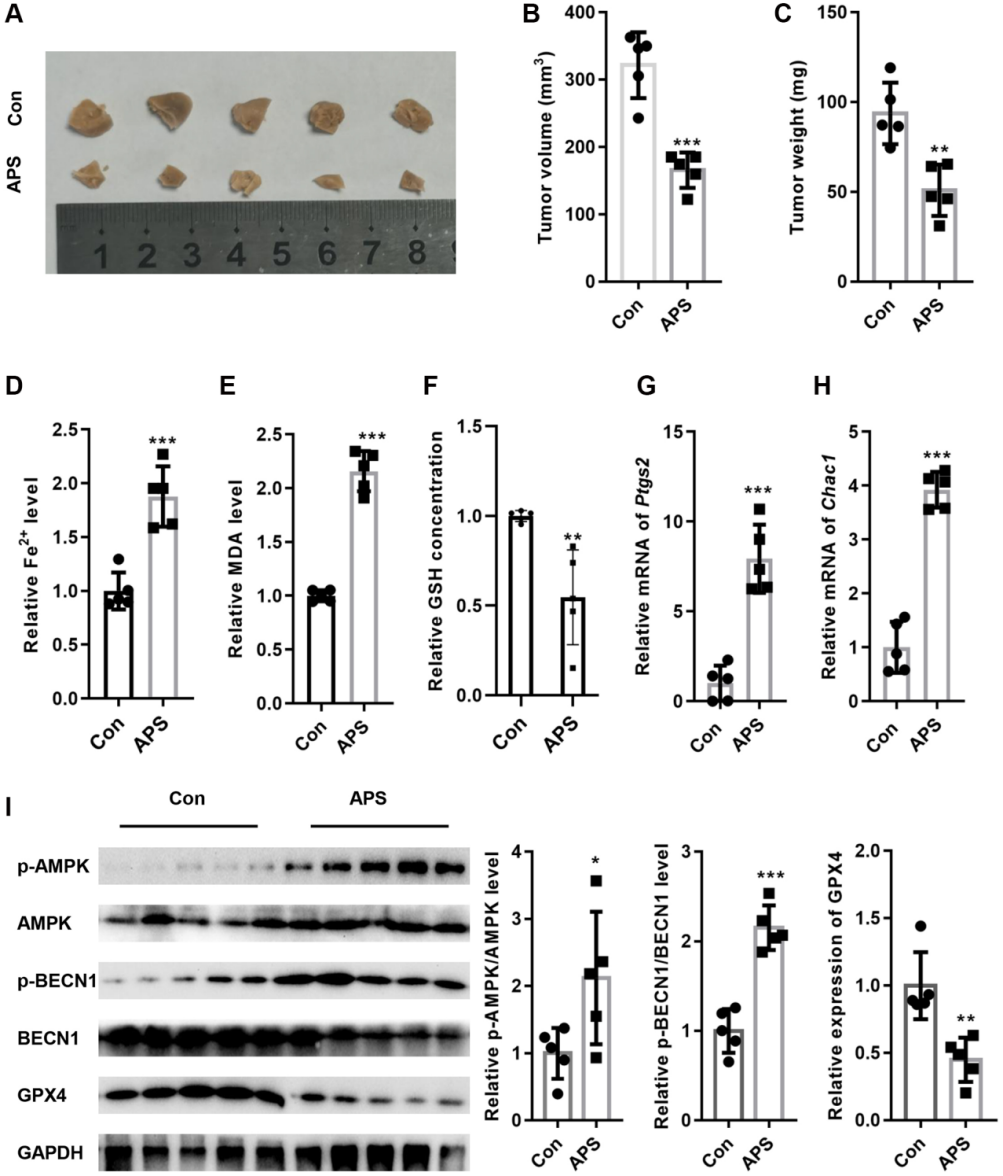
**APS inhibited the growth of tumors in nude mice.** (**A**) Representative images of tumor tissues from nude mice. APS significantly inhibited tumor volume (**B**) and weight (**C**) in nude mice. APS increased the levels of Fe^2+^ (**D**) and MDA (**E**) in tumor tissues. (**F**) APS decreased GSH levels in tumor tissues. The RT-PCR results showed that APS increased the mRNA levels of ptgs2 (**G**) and Chac1 (**H**) in tumor tissues. (**I**) After APS treatment, the phosphorylation levels of AMPK and BECN1 were increased in the tumor tissues of nude mice. ^*^p < 0.05, ^**^p < 0.01, ^***^p < 0.001 vs. Con.

Next, we examined the effect of APS on tumor growth in nude mice *in vivo*. As shown in Figure 7A–7C, APS significantly inhibited tumor volume and weight in nude mice. Moreover, APS increased the levels of Fe^2+^ and MDA in tumor tissues (Figure 7D, 7E). In addition, APS decreased the levels of GSH in tumor tissues (Figure 7F). APS increased the mRNA levels of the ferroptosis markers ptgs2 and Chac1 (Figure 7G, 7H). Furthermore, the phosphorylation levels of AMPK and BECN1 were increased in the tumor tissues of nude mice after APS treatment, as confirmed by Western blotting (Figure 7I). In contrast, APS treatment reduced the expression of GPX4 in the tumor tissues of nude mice (Figure 7H).

## DISCUSSION

In recent years, the incidence of UC has gradually been increasing, but the cure and survival rates of patients have not changed significantly [19]. Ferroptosis is a nonapoptotic, iron-dependent form of cell death that is activated in cancer cells by natural stimuli or synthetic drugs [20]. Ferroptosis occurs through multiple mechanisms: 1) increased intracellular iron levels; 2) direct inhibition of GPX4 expression; 3) inhibition of the cystine/glutamate transporter system Xc-; and 4) indirect inhibition of GPX4 activity, inhibition of GSH synthesis, inhibition of cystine entry into cells and the depletion of extracellular cysteine [20–22]. Currently, linking ferroptosis to key tumor suppressor pathways would be a strong breakthrough for precise cancer drug discovery. APS is one of the main active components of Astragalus, which can promote or modulate the immune response and has antiviral, antitumor, antiaging, and antioxidant effects. In the present study, we examined how APS inhibits the progression of UC for the first time.

We showed that APS inhibited the proliferation of RT4 and T24 cells in a concentration-dependent manner. Additionally, APS inhibited the migration of RT4 and T24 cells. We further examined the mechanism by which APS inhibits the proliferation and migration of RT4 and T24 cells, and CCK-8 analysis revealed that the ferroptosis inhibitor Fer-1 reversed APS-induced cell growth inhibition. Moreover, in RT4 and T24 cells, APS increased Fe^2+^ and MDA levels, whereas Fer-1 significantly reversed APS-induced Fe^2+^ and MDA accumulation. These results suggest that APS can trigger ferroptosis in UC cells.

Further analysis showed that APS significantly inhibited the expression of GPX4 but did not change the expression of xCT. Thus, we concluded that APS induced ferroptosis in RT4 and T24 cells, but whether it mediates ferroptosis in UC cells through system Xc-, a reverse transporter located at the plasma membrane and composed of xCT and SLC3A2, needs to be further investigated. xCT plays an important oncogenic role in the defense against oxidative stress and ferroptosis and in influencing malignant tumor behavior, the tumor microenvironment, the immune system, cancer-related symptoms and ferroptosis sensitivity [10, 23]. System Xc- transports glutamate extracellularly and cystine intracellularly. Then, cystine can be rapidly reduced to cysteine, which is used to synthesize GSH, an important antioxidant and free radical scavenger [24]. System Xc- can be inhibited by various ferroptosis inducers by reducing cystine uptake, which reduces GPX activity and ultimately leads to ferroptosis [15]. In the present study, we found that APS significantly suppressed GPX activity. Interestingly, we found that the activity of xCT was significantly inhibited.

Next, we examined the mechanism by which APS reduces the activity of xCT. Previous studies have shown that BECN1 is a key regulator of autophagy, and BECN1 protein expression was increased after treatment with the ferroptosis inducer erastin [15, 16]. BECN1 binds directly to the xCT protein to form a complex that inhibits xCT activity, which depends on AMPK-mediated phosphorylation of BECN1 at serines 90, 93 and 96 [16, 25]. In RT4 and T24 cells, APS significantly promoted AMPK-mediated BECN1 phosphorylation, which induced the formation of the BECN1-xCT complex to inhibit xCT activity, thereby triggering ferroptosis. To further verify that APS induces the formation of the xCT-BECN1 complex through AMPK-activated phosphorylation of BECN1, we screened an siRNA that specifically targeted AMPK and found that APS-induced ferroptosis was significantly reversed when AMPK was silenced in RT4 and T24 cells. These results suggest that AMPK-mediated formation of the BECN1-xCT complex is critical for APS-triggered ferroptosis. Next, we performed *in vivo* xenograft subcutaneous tumor experiments in nude mice. The *in vivo* results revealed that APS significantly suppressed the tumor volume and size. Moreover, the levels of Fe^2+^ and MDA in tumor tissues were significantly decreased after APS treatment. APS increased the mRNA levels of the ferroptosis markers ptgs2 and Chac1. At the protein level, APS increased the phosphorylation of AMPK and BECN1 in tumor tissues, and APS decreased the protein expression of GPX4 in tumor tissues. These *in vivo* results confirmed that APS inhibited UC progression by inducing ferroptosis.

However, there are limitations in the current study. Firstly, we did not explore the effect of APS on the long-term survival rate of tumor-bearing mice. Second, whether APS inhibits tumor growth through other mechanisms has not been reported. In addition, whether silencing BECN1 blocks the effect of APS has not been further explored.

**Figure 8:**
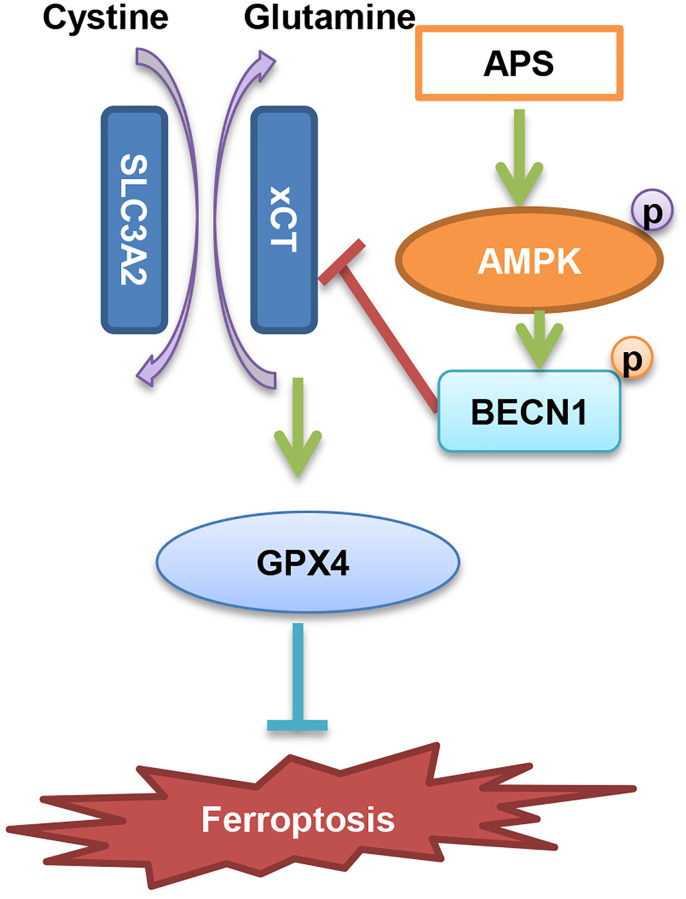
Diagram of the molecular mechanism by which APS inhibits UC.

In summary, we suggest that APS inhibits the progression of UC through the induction of ferroptosis. The specific mechanism is mainly dependent on AMPK activation-induced phosphorylation of BECN1, which then inhibits the formation of the BECN1-xCT complex and ultimately causes ferroptosis (Figure 8).

## MATERIALS AND METHODS

### Cell culture

The human urothelial cell lines RT4 and T24 were purchased from the American Type Culture Collection (ATCC^®^ HTB-2™, ATCC^®^ HTB-4™ and ATCC^®^ CRL-1573™, respectively). RT4 and T24 cells were cultured in DMEM (HyClone; GE Healthcare Life Sciences, USA) containing 1% penicillin-streptomycin solution (HyClone; GE Healthcare Life Sciences, USA) and 10% fetal bovine serum (FBS, HyClone; GE Healthcare Life Sciences, USA) at 37°C in a humidified incubator with 5% CO_2_.

### Cell counting Kit-8 (CCK-8) assay

RT4 and T24 cells were inoculated in 96-well plates at 5 × 10^3^ cells per well and incubated at 37°C until the monolayer was 70% confluent. After that, RT4 and T24 cells were incubated with Astragalus polysaccharide (APS, 89250-26-0, purity >98%, Nanjing Daosif Biotechnology Co., Nanjing, China) at concentrations of 0, 3.125, 6.25, 12.5, 25, and 50 and 0, 0.5, 1, 2, 5, 10, 20, and 30 μM. Cell viability was measured at 48 h according to the instructions of the CCK-8 kit (Beijing Solarbio Science and Technology Co., Ltd., China), and the optical density (OD) at 450 nm was measured and used to calculate the half-inhibitory concentration (IC50) of APS on the cells.

### Scratch test

RT4 and T24 cells were inoculated in a 6-well plate, and when the cells were a confluent monolayer, they were randomly divided into the Con and APS (15 and 10 μM) groups, with 3 replicate wells in each group. After that, the cells and debris were washed with PBS and treated with APS for 24 h. The cells were incubated at 37°C for 24 h. Cell migration was observed under a microscope at 0 and 24 h. Wound-healing rate = ((0 h scratch width - 24 h scratch width)/0 h scratch width) × 100%.

### Transwell chambers

RT4 and T24 cells were inoculated into the upper chamber of the Transwell (24 wells, Corning, USA) with a pore size of 8 μm (5 × 10^4^ cells per well, and 3 replicate wells for each group) in DMEM without FBS. The cells were treated with 15 and 10 μM APS at 37°C. The lower chamber contained 600 μL of DMEM containing 15% FBS and was incubated at 37°C for 24 h. After the cells penetrated the lower layer of the Transwell, the chamber was removed, and the medium was discarded. The cells in the upper chamber were gently wiped off with a cotton swab, fixed in 4% paraformaldehyde (Beijing Solarbio Science and Technology Co., Ltd., China) and incubated with 0.1% crystal violet (Beijing Solarbio Science and Technology Co., Ltd., China) for 30 min at room temperature. Finally, five randomly selected fields were photographed under a microscope (×20) to observe cell migration.

### Measurement of intracellular iron levels

RT4 and T24 cells were inoculated in 6-well plates at a density of 2 × 10^5^/well and incubated at 37°C for 24 h. Afterward, RT4 and T24 cells were treated with 15 and 10 μM APS for 24 h. Intracellular iron levels were measured using an Iron Assay Kit (ab83366, Abcam, UK). The assay was performed in accordance with the instructions. The absorbance of each well at 450 nm was measured using an ELISA microplate reader (ELx800, BioTek, USA).

### Malondialdehyde (MDA) assay

RT4 and T24 cells were inoculated in 6-well plates at a density of 2 × 10^5^/well and incubated for 24 h. RT4 and T24 cells were then treated with 15 and 10 μM APS. Intracellular MDA levels were determined using the Lipid Peroxidation (MDA) Assay Kit (ab118970, Abcam, UK). The absorbance of each well was measured at 532 nm using an ELISA microplate reader (ELx800, BioTek, USA).

### Reduced glutathione (GSH) assay

RT4 and T24 cells were inoculated in 6-well plates at a density of 2 × 10^5^/well and incubated for 24 h. RT4 and T24 cells were then treated with 15 and 10 μM APS for 24 h. Intracellular GSH levels were measured using GSH and GSSG assay kits (Beyotime, Beijing, China), and the absorbance of each well at 420 nm was measured using an ELISA microplate meter (ELx800, BioTek, USA).

### RT-PCR

RT-qPCR was used to determine prostaglandin-endoperoxide synthase 2 (ptgs2) and chaC glutathione specific gamma-glutamylcyclotransferase 1 (chac1) mRNA levels in RT4 and T24 cells, and RNA was extracted from each group of cells according to the SuperScript IV reverse transcription kit (Invitrogen; Thermo Fisher Scientific, Inc., Waltham, MA, USA). The mRNA levels of ptgs2 and chac1 in each group of cells were measured by a TaqMan real-time fluorescence PCR kit (Invitrogen; Thermo Fisher Scientific, Inc., Waltham, MA, USA). The reaction system was as follows: 9.5 μL RNase Free dH2O, 1 μL cDNA/DNA, 1 μL upstream primer, 1 μL downstream primer, and 12.5 μL 2× UltraSYBR Mixture. GAPDH was used as an internal control. The primer sequences were as follows:

ptgs2-F: GAGGGATCTGTGGATGCTTCG;
ptgs2-R: AAACCCACAGTGCTTGACAC;
chac1-F: CTCAGCCCAGCCATCCATAG;
chac1-R: CAAGTGGGTAAGAGGCCCAG;
GAPDH-F: TGACCACAGTCCATGCCATC;
GAPDH-R: TCCTCTTGTGCTCTTGCTGG.

### Western blotting

RT4 and T24 cells were obtained by adding precooled RIPA lysis buffer (Beijing Solarbio Science and Technology Co., Ltd., China), the samples were centrifuged at 4°C for 15 min, and the supernatant was collected. The protein concentration was determined by using a BCA kit (Beijing Solarbio Science and Technology Co., Ltd., China and mixed with 1/4 volume of protein loading buffer (Beijing Solarbio Science and Technology Co., Ltd., China) at 100°C for 5 min. The proteins were separated by electrophoresis using 10% SDS-PAGE. The membranes were incubated with primary antibodies against p-AMPK (Thr172; 50081; 1:1,000; Cell Signaling Technology, Inc., Danvers, MA, USA), AMPK (5831; 1:1,000; Cell Signaling Technology. Inc., Danvers, MA, USA), p-BECN1 (Ser93, 14717; 1:1,000; Cell Signaling Technology, Inc. Technology, Inc., Danvers, MA, USA), GPX4 (59735; 1:1,000; Cell Signaling Technology, Inc., Danvers, MA, USA), xCT (12691; 1:1,000; Cell Signaling Technology, Inc., Danvers, MA, USA), and GAPDH (5174; 1:1,000; Cell Signaling Technology, Inc., Danvers, MA, USA) overnight at 4°C. Then, the membranes were incubated with the secondary antibody (Beijing Solarbio Science and Technology Co., Ltd., China) at a ratio of 1:5000 for 1 h at room temperature. Then, the membranes were washed three times with TBST for 15 min each time. The relative expression of each target protein was calculated by ImageJ 1.43b software (National Institutes of Health, Bethesda, MD, USA), and GAPDH was used as the internal reference.

### Flow cytometry

RT4 and T24 cells were inoculated in 6-well plates at a density of 2 × 10^5^/well and incubated for 24 h. RT4 and T24 cells were then treated with 15 and 10 μM APS for 24 h. Then, the cells were collected, and cell death was determined using an Annexin V-PE/7-AAD Apoptosis Detection Kit (Meilunbio Co. Ltd., Dalian, China). The cells were washed once with PBS, washed once with 1× binding buffer, and resuspended in a volume of 100 μl. Then, the cells were incubated with 5 μl Annexin V for 15 min at room temperature and then 5 μl of 1× 7-AAD for 10 min at room temperature. The cells were detected on a CytoFLEX flow cytometer (Beckman Coulter) and the data was analyzed using FlowJo V10 software (Shanghai, China). On the scatter plot of the bivariate flow cytometer, normal cells (AnnexinV-/7-AAD-) were in the lower left quadrant, early apoptotic cells (AnnexinV+/7-AAD-) were in the lower right quadrant, and late apoptotic cells (AnnexinV+/7-AAD+) were in the upper right quadrant.

### Transient transfection

The specific siRNA oligonucleotide sequence targeting AMPK was 5′-AAAGTGAAGGTTGGCAAACATGA-3′; the negative control was 5′-UUCUCCGAACGUGUCACGUTT-3′. The specificity of the siRNA oligonucleotide sequence was determined by nucleotide BLAST comparison with all other sequences in GenBank. siRNA transfection was performed using HiPerFect Transfection Reagent (Qiagen, Germany) according to the instructions. For the experiments, RT4 and T24 cells were cultured in six-well plates. Then, 12 μl of HiPerFect Transfection Reagent, 500 ng of siRNA or negative control (NC) RNA, and 100 μl of serum-free DMEM were mixed and added to the wells after standing for 10 min at room temperature.

### Lipid peroxidation assay

RT4 and T24 cells were inoculated into 6-well plates and treated with APS for 24 h. After that, the lipophilic fluorescent dye C11 BODIPY 581/591 (1 μM, Shanghai Maokang Biotechnology Co., Ltd., Shanghai, China) was added to RT4 and T24 cells, incubated at 37°C for 30 min and observed under a fluorescence microscope (Keyence, Japan).

### FerroOrange staining

RT4 and T24 cells were inoculated into 6-well plates and treated with APS for 24 h. After that, FerroOrange fluorescent dye (2 μM, HY-D1301, MCE, USA) was added to RT4 and T24 cells, incubated at 37°C for 30 min and observed under a fluorescence microscope (Keyence, Japan).

### Immunofluorescence staining

Cells were fixed with 4% paraformaldehyde (Beijing Solarbio Science and Technology Co., Ltd., China) at room temperature for 15 min. Subsequently, the cells were washed twice with PBS, permeabilized with 0.4% Triton-X100 (Beijing Solarbio Science and Technology Co., Ltd., China.) for 30 min and blocked with 5% BSA at room temperature for 1 h. The cells were then incubated with primary antibodies against xCT/BECN1/GPX4 at a ratio of 1:100 at 4°C overnight and with secondary antibodies at room temperature for 1 h. The cells were then incubated with DAPI (Beijing Solarbio Science and Technology Co., Ltd., China) for restaining. Images were acquired using fluorescence microscopy and analyzed using ImageJ 1.43b software (National Institutes of Health, Bethesda, MD, USA).

### JC-1 staining

Cells were treated as described above. Then, the cells were washed with PBS and stained with 20 μM JC-1 probe (C2006, Beyotime, Beijing, China) at six-well plates for 10 min and observed under a fluorescence microscope immediately.

### Glutamate release assay

Glutamate release rate was determined using the Amplex Red Glutamate Release Assay Kit (Thermo Fisher Scientific, A12221) according to the instructions.

### Nude mouse tumorigenesis experiment

A total of 10 male nude mice (aged 6~7 weeks) were purchased from Shanghai Jieshige Experimental Animal Co. The mice were housed in a specific pathogen-free (SPF) environment in the central laboratory of Jinzhou Medical University. The experimental design was approved by the Animal Experimentation Ethics Committee of Jinzhou Medical University (Grant No. 2022-053). The mice were fed normal chow, had free access to food and adequate water, and were maintained under a 12 h/12 h light/dark cycle at 24°C with a relative humidity of 40–70%.

RT4 cells in the logarithmic growth phase were washed with sterile PBS and centrifuged at 1,000 r/min (5 min). The supernatant was discarded, and the cells were washed again with sterile PBS and finally resuspended in PBS. After that, 0.1 mL of the cell suspension (2 × 10^6^ cells) was slowly injected into the dorsal subcutis of the mice. After 7 days, the mice were randomly divided into 2 groups, the Con group and the APS group (*n* = 5 for each group). The nude mice in the APS group were administered 5 mg/kg APS by gavage, and the nude mice in the Con group were administered the same amount of saline once every 3 days. The longest diameter a (mm) and the shortest diameter b (mm) of the tumor were measured once per week, and the tumor volume was determined as follows: V = a × b^2^ × 0.52 (mm^3^). Mice were anesthetized using 2–3% inhaled isoflurane and the depth of anesthesia was monitored by toe pinch response. The nude mice were sacrificed on Day 21, and the tumor mass was measured.

### Statistical analysis

The data were processed using SPSS 25.0 statistical software. p-P plots were used to test the normality of the data, and measures that conformed to a normal distribution are expressed as × ± s. Independent samples *t* tests were used to compare two groups, and one-way ANOVA followed by *post-hoc* analysis was used to compare multiple groups. A *p* < 0.05 was statistically significant.
